# Goiter and thyroid eye sign in the Madonna and Child with Angels by Francesco del Cossa (c. 1430–1477)

**DOI:** 10.1007/s40618-023-02012-8

**Published:** 2023-01-14

**Authors:** H. Ashrafian

**Affiliations:** Institute of Global Health Innovation, The Department of Surgery and Cancer, Imperial College London, St Mary’s Hospital, 10th Floor Queen Elizabeth the Queen Mother (QEQM) Building, Praed Street, London, W2 1NY UK

The School of Ferrara represented a unique blend of North Italian artistic styles during the Renaissance, taking influence from, and eventually contributing to, the art in Florence, Bologna, Lombardy, Mantua and Venice. Among its most prominent artists was Francesco del Cossa (c. 1430–1477) whose ability stretched from painting, stone masoning (apprenticed form his father) and stained-glass work.

**Fig. 1:**
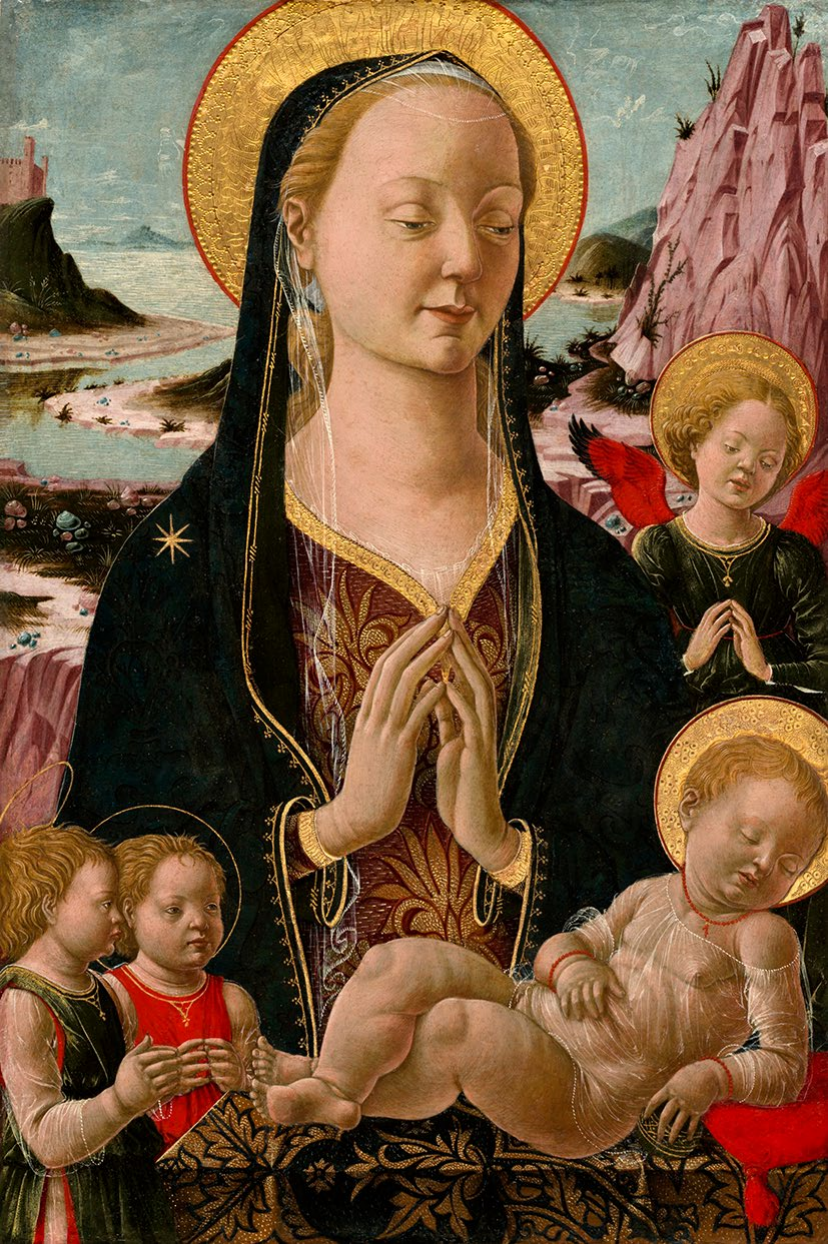
Francesco del Cossa, Madonna and Child with Angels (c. 1455/1470), tempera on poplar panel; dimensions: overall: 53.5 × 36.2 cm (21 1/16 × 14 1/4 in.) framed: 68.6 × 51.4 × 7 cm (27 × 20 1/4 × 2 3/4 in.) © National Gallery of Art, Washington, D.C., USA

I note that his 1470 painting entitled ‘Madonna col Bambino e Angeli’ (Fig. 1) demonstrates the main Madonna figure to have a notable Goiter. There is additional evidence of eye involvement with infra-orbital swelling consistent with inferior-eye Enroth Sign [1].

The differential diagnosis for this Goiter with thyroid eye involvement at this time in Renaissance Italy would most likely derive from autoimmune thyroiditis with subclinical thyroid failure due to the absence of generalized myxedema. This is because there is no evidence of (a) overall hyperthyroidism (prevalence around > 1% in the general population and is relatively infrequent in iodine-deficiency areas) and no evidence of specific (b) Graves’ orbitopathy (GO) as the most frequent extra-thyroidal expression of Graves’ disease (in approximately 25–30% of patients). The term ‘Thyroid Eye Disease’ is usually associated with Graves’ hyperthyroidism which is not clinically represented here, rather there is hypothyroid-associated eye involvement. Furthermore, (c) endemic iodine deficiency (well established in Renaissance Italy) is also unlikely as this typically results in endemic Goiter (diffuse and/or nodular), congenital and acquired hypothyroidism, various degrees of mental retardation or neurological development and growth impairment and occasionally endemic cretinism, though eye involvement is rare. The ‘closed eye’ ptosis image is likely an artistic stylism well stablished in that era. Hypothyroidism typically only represents up to 10% of individuals with thyroid eye signs [2], though with these features is the most likely diagnosis by autoimmune Hashimoto thyroiditis.

Francesco del Cossa was a keen artist of frescoes and demonstrated an ability to focus on eye paintings [3] in subsequent works, and left Ferrara for financial reasons to go to Bologna, eventually succumbing to Bubonic plague. This work emphasizes his ability to capture the tangibility of clinical signs in his artistic impression and adds to the corpus of understanding disease trends [4] during the Renaissance era while highlighting the ability of the master artists of the time.
